# Realizing Virtual Care in VA: Supporting the Healthcare System’s Journey Towards Enhanced Access, Engagement, and Outcomes

**DOI:** 10.1007/s11606-024-08618-9

**Published:** 2024-02-23

**Authors:** Timothy P. Hogan, Scott E. Sherman, Navid Dardashti, Nicholas McMahon, Cindie Slightam, Donna M. Zulman

**Affiliations:** 1 Center for Healthcare Organization and Implementation Research (CHOIR), VA Bedford Healthcare System, Bedford, MA USA; 2 Peter O’Donnell Jr. School of Public Health, UT Southwestern Medical Center, Dallas, TX USA; 3 VA New York Harbor Healthcare System, New York, NY USA; 4 Department of Population Health, NYU Grossman School of Medicine, New York, NY USA; 5 VA Center for Innovation to Implementation (Ci2i), Menlo Park, CA USA; 6 Division of Primary Care and Population Health, Stanford University School of Medicine, Stanford, CA USA

In 1959, the Nebraska Psychiatric Institute implemented a two-way television system to conduct group therapy with Veterans located at the Omaha Veterans Health Administration (VHA) Medical Center and other VHA facilities, launching VHA’s journey towards virtually delivered healthcare^1^. In the intervening decades, VHA has developed and adopted an ever-evolving suite of technologies intended to facilitate remote monitoring and disease management, enhance Veteran self-management, support patient communication with their care team, and host real-time remote clinical appointments across disparate locations. Led by VHA’s Office of Connected Care (OCC), the entity responsible for the healthcare system’s digital health strategy, VHA has adopted the term “virtual care” to refer to such technologies, with the goal of enhancing the accessibility, capacity, quality, and experience of VHA healthcare for Veterans, their families, and their caregivers, wherever they are located^2^.

Like healthcare systems throughout the world, VHA’s virtual care journey was accelerated in 2020 when the COVID-19 pandemic took hold. Driven by the need to provide Veterans with access to VHA services while reducing their risk of infection from a novel coronavirus, there was a 1000% surge in the use of synchronous video visits^3^, while the ongoing use of other technologies (e.g., the MyHealth*e*Vet patient portal) supported asynchronous communication between Veterans and their clinical teams and critical functions like medication refilling^4^. Although uptake in use was a defining feature of the pandemic, the story was not that linear; surges in virtual care were not uniform across VHA’s suite of technologies, and beyond VHA, the proliferation of telehealth illuminated important disparities in technology-related access and implications for care delivery. Nationwide, policy questions were raised about virtually﻿-delivered healthcare services across state lines, healthcare providers were confronted with new ways of delivering care that presented profound workload implications, and care processes often had to be completely reinvented.

While the pandemic rapidly shifted virtual care modalities to the center of healthcare discussions, there remain longstanding issues surrounding virtual care that demand attention. These include the proliferation of virtual care technologies within and outside VHA, the potential of those technologies to address disparities in care but to inadvertently exacerbate others, generational shifts in patient preferences for the delivery and receipt of healthcare, and an evolving but limited evidence base on the impacts of virtual care and the contexts in which it is most effectively utilized. Taken together, these developments underscored the need to convene experts in virtual care to assess the state of the related science and to articulate a guiding research agenda for VHA. The establishment of VHA’s Virtual Care Consortium of Research (VC-CORE) in 2020, with its goals to facilitate the adoption and use of virtual care, foster virtual care-related research aligned with healthcare system priorities, and build a network of investigators to pursue that research, was the catalyst to plan a VHA Virtual Care State of the Art (SOTA) conference intended to address these needs.

The Virtual Care SOTA was held over a day and a half in New Orleans in May 2022. Preparations for the event were made over the prior year and were overseen by the VC-CORE in conjunction with national research leadership. The principal investigators of the VC-CORE formed a conference planning committee comprising investigators with diverse research interests and subject matter expertise. Planning activities also involved OCC leadership and Veteran representatives to ensure that the conference topics and activities aligned with VHA’s strategic needs and addressed Veterans’ priorities. Planning activities were also anchored by a process-oriented perspective on the use of virtual care, namely that Veterans must first gain access to virtual care technologies, subsequently adopt and sufficiently engage with the technologies as part of their care, and in so doing, possibly realize improved outcomes. The planning committee captured this perspective in a continuum of Access, Engagement, and Outcomes that came to provide an overall framework (Fig. 1) for the SOTA’s focus.

**Figure 1:**
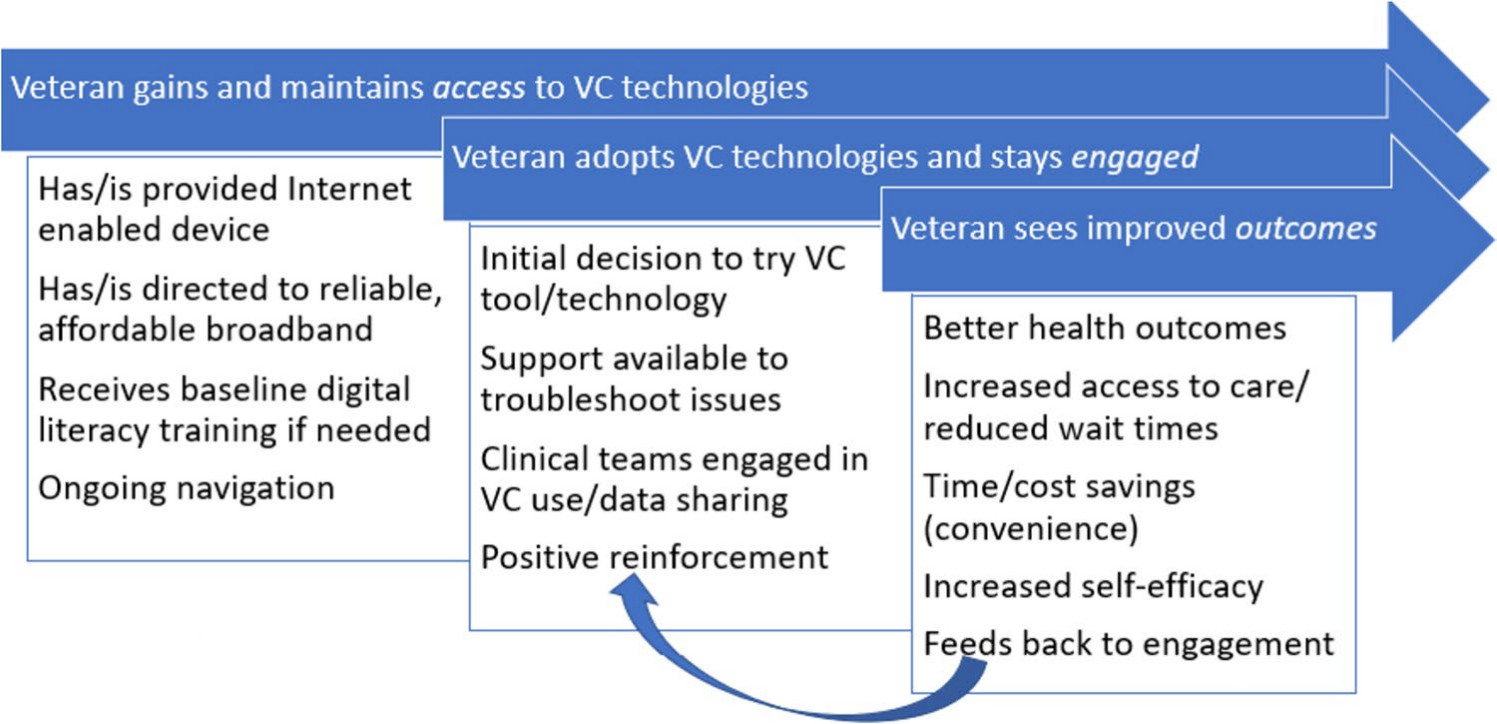
Continuum of access, engagement, and outcomes.

With this framework for guidance, the planning committee created three workgroups, each charged with identifying research priorities related to one of the framework components: (1) address virtual care access disparities, (2) enhance Veteran engagement with virtual care, and (3) define and improve outcomes influenced by virtual care. The focus of these three workgroups also aligned directly with the mission of VHA’s OCC, to enhance the healthcare experience through virtual care technologies, including the delivery of high-quality, Veteran-centered care, optimized individual and population health, and care that is personalized and proactive^2^.

The conference opened with a plenary session and quickly pivoted to attendees gathering in assigned workgroups for structured discussion sessions. These sessions focused on the identification and prioritization of research questions, which are the subject of three papers in this special issue. On the second day of the conference, each of the three workgroups presented the research questions they identified to all attendees. SOTA attendees then participated in a prioritization exercise in which they selected a total of five research questions from across the workgroups that they viewed as most important for future VHA research focused on virtual care. As shown in Table 1, these priorities are cross-cutting and address diverse topics related to virtual care access, engagement, and outcomes that could be pursued as part of a research agenda with short-, intermediate-, and long-term goals.

**Table 1:** Top 5 Virtual Care SOTA Research Priorities

Research priority	Votes
Identify which implementation strategies increase patient/clinician adoption of effective virtual care technologies	23
Determine the optimal care portfolio (i.e., mix of in-person and virtual care encounters) to create the “best” outcomes overall	17
Understand the Veteran journey, from the period of active service to the period of being a VA patient, and when and how virtual care can best be introduced along that journey to maximize engagement	16
Identify and evaluate opportunities to optimize Veterans’ access to virtual care through interventions at the patient, provider, system levels	14
Examine how best to use patient-generated health data (PGHD) in combination with traditional sources of data, such as the electronic medical record (EMR), to generate alerts and predictions that are clinically valuable to providers	14

Since the conclusion of the SOTA, the VC-CORE has disseminated the research priorities identified at the conference to its national network of investigators and to VHA more broadly, through a workshop at the 2023 HSR&D/QUERI National Meeting and through Cyberseminars, newsletters, and VHA’s intranet. Efforts are also underway, in partnership with research and OCC leadership, to develop targeted requests for applications (RFAs) and other funding opportunities to directly address these priorities. These priorities are also reflected in the two perspectives pieces, as well as the 12 original research manuscripts previewed below, that comprise this special issue and which map to the broad domains of virtual care access, engagement, and outcomes.

## VIRTUAL CARE ACCESS

Five papers in this issue focus on opportunities to expand access to VHA services via virtual care. Schubert et al. describe the process of adapting an in-person home-based geriatrics program into a hybrid-virtual model that offers community-dwelling older Veterans access to home visits. Their paper highlights challenges of using telehealth with Veterans who have complex medical needs, such as conditions requiring a physical exam^5^. Two other papers examine how the COVID-19 pandemic shaped patterns of virtual care utilization. Lum et al. examine growth in telehealth use for mental healthcare between 2019 and early 2023 and find that a striking 54% of Veterans in their cohort relied exclusively on video and phone and that wait times for in-person and virtual appointments were equivalent^6^. Leung et al. assess use of video-based primary care among homeless-experienced Veterans before and during the pandemic and describe how the proportion of Veterans who had a video-based visit in primary care increased dramatically from 1 to 21%^7^. Tisdale et al. characterize use of VHA video primary care for Veterans with cardiovascular diseases, finding lower use among specific subgroups, suggesting possible disparities and needs for targeted supports^8^. Lastly, Javier et al. describe persistent racial-ethnic disparities in the context of telehealth specialty pain care during the COVID-19 pandemic, advocating for additional research to examine how such disparities could be reduced moving forward^9^.

## VIRTUAL CARE ENGAGEMENT

Four papers in this issue speak to Veteran engagement with virtual care technologies. Robinson et al. present survey data regarding Veteran adoption of digital health devices (DHDs), finding that the vast majority of Veterans in their cohort were current or past users of DHDs, including lifestyle monitoring and self-management devices^10^. McLaughlin et al. report on their efforts to test the effect of mailed informational postcards on remote monitoring adherence among Veterans with pacemakers and implantable cardioverter-defibrillators, finding that simple outreach can substantially impact engagement^11^. Peracca et al. describe a theory-driven evaluation of VHA’s efforts to roll out direct-to-Veteran asynchronous teledermatology, capturing the variation in experiences across participating VHA facilities^12^. Finally, El Shahawy and colleagues share survey findings underscoring that various reasons may underlie Veteran visit modality preferences, and posit that such preferences can inform the delivery of Veteran-centered care^13^.

## VIRTUAL CARE OUTCOMES

Within this issue, three papers focus on the outcomes of virtual care. Across a 2-year study period which included a year during the COVID-19 pandemic, O’Shea et al. found that Veterans who had at least one video visit were more likely to have subsequent emergent care and hospitalization, and this also included hospitalization for ambulatory care–sensitive conditions (such as an asthma exacerbation)^14^. VHA has established Clinical Resource Hubs to facilitate national delivery of care via telehealth, and Gujral et al. found that sites with these hubs had higher rates of delivering telephone primary care visits and lower rates of emergent care and hospitalization during the pandemic than sites without a hub^15^. In the final paper, Midboe et al. studied the relationship between patient portal use and medication adherence and viral load in Veterans living with HIV^16^. They found that during a 4.5-year period prior to the COVID-19 pandemic, both refilling a prescription through the patient portal and sending a secure message were associated with better adherence to antiretroviral medications, while viewing lab results through the portal was associated with better viral suppression.

## CONCLUSION

As a healthcare system that serves over 5 million patients per year and maintains a robust and comprehensive electronic health record, VHA has an opportunity to lead the nation in further generation of evidence about virtual care’s potential impact, and to explore more nuanced questions regarding its optimal use. VHA’s Virtual Care SOTA revealed the breadth of research topics that demand attention across the domains of access, engagement, and outcomes. The papers in this special issue illustrate the high-quality research that is already emerging from VHA’s network of virtual care researchers, as well as the important work still to do to support the realization of virtual care in VHA.
